# A Case of Unifocal Eosinophilic Granuloma of the Mandible in an Adult Female: A Case Report

**DOI:** 10.1155/2012/521726

**Published:** 2012-08-16

**Authors:** Anshita Agarwal, Gaurav P. Agrawal, Sarwar Alam, Benazeer Husain

**Affiliations:** ^1^Department of Oral Pathology and Microbiology, Career Post Graduate Institute of Dental Sciences & Hospital, Lucknow 226020, India; ^2^Department of Oral Pathology and Microbiology, Vasant Dada Patil Dental College, Sangli 416306, India; ^3^Department of Oral and Maxillofacial Surgery, Career Post Graduate Institute of Dental Sciences & Hospital, Lucknow 226020, India

## Abstract

Eosinophilic granuloma of bone is a disease with an incidence of one new case per 350,000 to 2 million per year, which is an uncommon disease of maxillofacial region, and presents in more than 90% in children under the age of ten with predominance for males. As a result, eosinophilic granuloma of the jaw is always unconsidered in the differential diagnosis of similar lesions by many clinicians. It is difficult to make a correct diagnosis on it without proof of a pathological diagnosis, which correlates with the diverse clinical and radiographic presentations of eosinophilic granuloma in the jaws. In the present paper we report a rare case of unifocal eosinophilic granuloma of mandible occurring in an adult female.

## 1. Introduction

The term “Eosinophilic Granuloma of bone” was introduced by Lichtenstein and Jaffe in 1940 [1]. Eosinophilic granuloma is one of the rarest bone tumors representing less than 1% of them. In 90% of the reported cases it appears in children under the age of ten. There is a certain predilection to males in the ratio of 2 : 1 [2]. It is a localized and mildest form of histiocytosis-X group of diseases, which also encompasses Hand-Schuller-Christian syndrome and Letterer-Siwe syndrome. The above grouping has been based on the similarities of the histopathologic appearance of the histiocytic and eosinophilic proliferation.

Eosinophilic granuloma may not present physical signs or symptoms in the clinical observation and most of the times it is discovered during routine radiographic examination. Sometimes there may be localized swelling, paing or tenderness. The lesion may occur in the jaw and overlying soft tissues of the mouth although the skull and mandible are common regions of involvement, the femur, ribs, humerus, and other bones may also be affected. Loss of superficial alveolar bone and localized periodontitis are common early forms of the disease. unifocal eosinophilic granuloma of bone has a destructive nature, and is well demarcated, roughly round or oval in shape. The area destroyed replaced by a soft tissue (brown in color) since there is no necrosis, later the lesion becomes fibrous and grayish.

Under the direction of the Writing Group of the Histiocyte Society, Langerhans cell histiocytosis has been adopted as the appropriate clinicopathologic designation that encompasses and essentially replaces the previous historical terms used to classify this category of abnormal histiocyte proliferation [3]. However, many authors continue to utilize the term eosinophilic granuloma of bone; thus, this latter terminology is still both historically and clinically relevant.

## 2. Case Report 

A 35-year-old female reported to the out-patient department, Career Post Graduate Institute of Dental Sciences & Hospital, Lucknow on January 3rd, 2011 with a complain of unhealed dental socket present on the right-posterior mandibular alveolar ridge. 6 weeks agothe patient visited a local dentist with complain of loose teeth, diagnosis of periapical pathology was made and she got her few teeth removed. Antibiotics and anti-inflammatory drugs where prescribed for 5 days. After 2 weeks the patient started experiencing dull continuous pain in the same region with salty taste sensation and foul smelling odor from mouth, for which she again went to the same dentist, previously prescribed drugs were repeated and betadin mouthwash was also recommended. The patient found no improvement so she decided to consult our institute. The physical and neurological examination had no pathological signs. No fever, elevated regional temperature, or lymphadenopathy were recorded with no history of diabetes,numbness, or weakness. Intraoral examination revealed unhealed sockets in respect to 46, 47 and 48. No hemorrhagic areas or purpura was observed. The differential diagnosis include osteomyelitis, Paccioni granulations, Ewing's sarcoma, osteosarcoma, multiple, myeloma and metastasis. 

**Figure 1:**
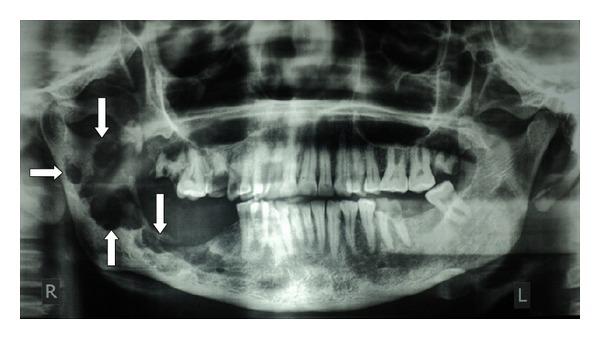
Orthopantamograph showing radiolucent area with irregular borders with no evidence of calcification or ossifications (arrows).

Orthopantomograph demonstrated round/ovoid punched out lesion with beveled edge = hole-within-a-hole appearance extending from premolar area up to the right condyle. Its borders were irregular and no calcification or ossification in the lesion was noted (Figure 1). In full body CT scan no secondary or multiple foci were enhanced. 

Blood examination revealed no pathological findings, neither of elevation of leukocytes nor of erythrocyte sedimentation rate. Heart ultrasound was made which revealed no pathologic findings.

**Figure 2:**
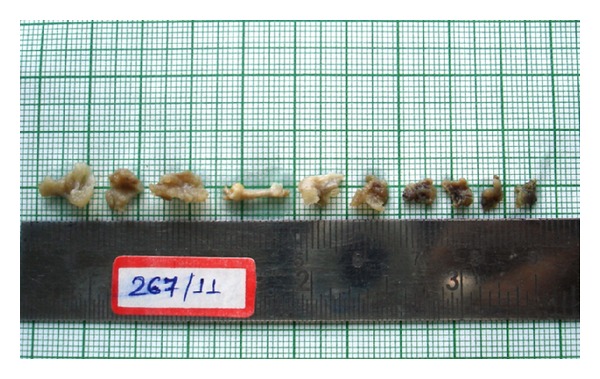
Multiple small bits of soft and hard tissue with ill-defined borders and yellow-tan in appearance.

**Figure 3:**
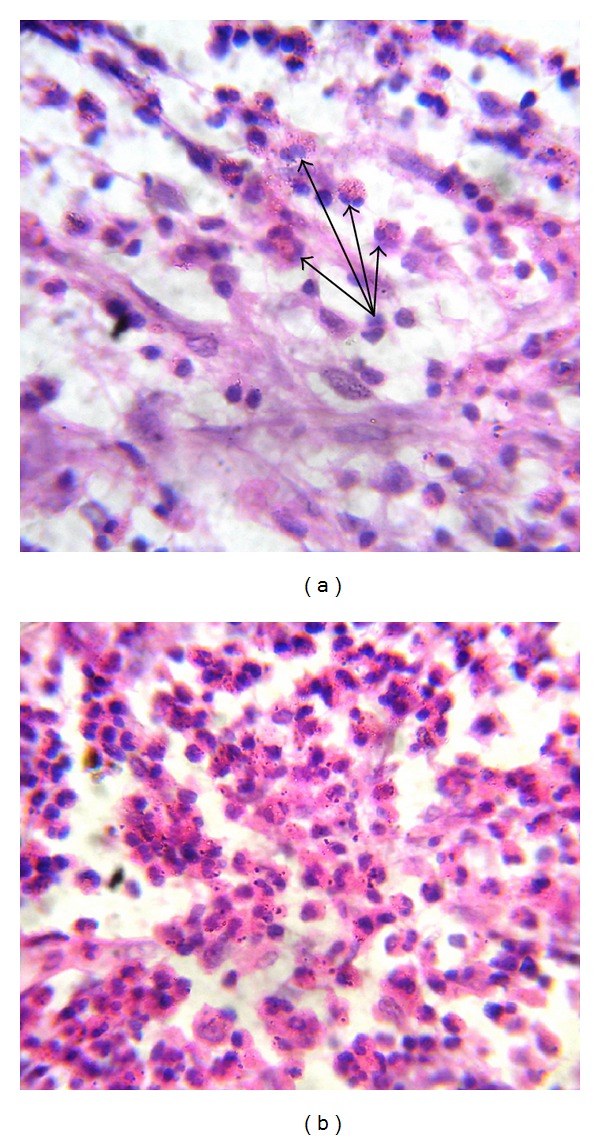
(a) Photomicrograph showing an admixture of inflammatory cells, including occasional Eosinophils (arrows). (H&E, ×100). (b): Photomicrograph showing clusters of large lesional cells with abundant bright pink cytoplasm and reniform nucleus. (H&E, ×100).

Surgical curettage was done and bone graft was placed under local anesthesia and samples were sent to the department of oral pathology and microbiology for the histopathological diagnosis. Macroscopically, the gross has small, multiple masses of both hard and soft tissue measuring less than 1 cm. Soft tissue masses were granular and gelatinous, appearing gray red to brown with flecks of yellow, while bony masses appeared yellow-tan with ill-defined borders (Figure 2). Microscopically, there were areas of histiocytic proliferation with focal collection of eosinophils (Figures 3(a) and 3(b)). Loose connective tissue stroma showed numerous suspended neutrophils and small mature lymphocytes. 

Postoperative outcome of lesion was uneventful and showed good prognosis. After 20 weeks removable prosthesis was given and no recurrence was observed till date.

## 3. Discussion 

Eosinophilic granuloma usually presented as a monostotic lesion affecting flat and long bones (70%) [4], the skull bone, jaw bone, and the vertebral spine. A unique cell, the Langerhans cell is diagnostic which was derived from the mononuclear cell and dendritic line precursors and is found in the bone marrow [5]. The cell is mainly identifiable under the electron microscope as it has racket-shaped cytoplasmic inclusion known as Birbeck granules. The pathognomonic cell, the Langerhans cell, excretes IL-1, and PG-E2 as to damage surrounding tissues. Though not a proven affiliation [6], eosinophilic granuloma can be asymptomatic or present as local swelling, pain, or tenderness. Depending on the location of the tumor, it may cause neurological symptoms such as numbness, limping, fracture, loosening of teeth, otitis media [7], or exopthalmos. If it involves skull, then a hematoma after a mild injury is a common finding [8]. Eosinophilic granuloma is clinically often confused with focal infection, since patients may have a slight fever, elevated sedimentation, and mild leukocytosis. Approximately 10% of patients with unifocal eosinophilic granuloma of bone will develop multifocal and extraosseous disease. In the present case no fever or other signs of inflammation have been reported it may be because the patient has already taken a heavy dose of antibiotics and anti-inflammatory drugs and blood examination also revealed no pathological findings.

On plain radiographs, eosinophilic granuloma typically presents as a punched out lesion with reactive sclerosis. A less common finding is one of a permeative pattern with or without periosteal reaction. The calvaria and especially the parietal bones are most often affected, followed by the mandible, the ribs, and the pelvis. Lesions in the long bones are most often in the diaphysis (58%). Epiphysis is rarely involved (2%). Lesions in the calvaria are between 1 to 15 cm in diameter. In patients with systemic disease, lesions are generally larger and can measure up to 25 cm [9]. 

The tumor material is sterile but there have been reports about the presence of staphylococcus and streptococcus [10]. Eosinophils, lymphocytes, fibroblasts, and foam cells may also be found but none of them is pathognomonic but they are suggestive of diagnosis. These lesions when associated with langerhans cells, called as Langerhans cell histiocytosis. Lineage which leads to formation of histocytes from Langerhans cells is debatable but was found to be associated [11]. Thus, presence of histiocytes alone with eosinophils has a diagnostic value. The only reliable immunological marker is the OKT6 while the common S-100 protein is usually positive too.

Eosinophilic granuloma does not lead to malignant transformation. If it expands elsewhere but bones then is called Hand-Schuller-Christian disease and may manifest as diabetes insipidus, cerebellar, hypothalamic, and with other central nervous system symptoms. The prognosis depends on the age of diagnosis and the number of foci.

In general, no treatment is needed for localized osseous eosinophilic granuloma and often the biopsy is enough to initiate healing. Steroid injection, curettage, excision, or radiation may be necessary depending on the extent of the disease and the symptoms.

## 4. Conclusion

This is one of the rare cases of eosinophilic granuloma of the mandible developing in an adult female patient. It is important to include eosinophilic granuloma in the differential diagnosis of bone lesions in adult subjects because of the possible expansion of the disease if untreated. We suggest a followup of a year is necessary for probable recurrence.
